# Synchrony between orientation-selective neurons is modulated during adaptation-induced plasticity in cat visual cortex

**DOI:** 10.1186/1471-2202-9-60

**Published:** 2008-07-03

**Authors:** Narcis Ghisovan, Abdellatif Nemri, Svetlana Shumikhina, Stephane Molotchnikoff

**Affiliations:** 1 Department of Biological Sciences, University of Montreal, QC, Canada

## Abstract

**Background:**

Visual neurons respond essentially to luminance variations occurring within their receptive fields. In primary visual cortex, each neuron is a filter for stimulus features such as orientation, motion direction and velocity, with the appropriate combination of features eliciting maximal firing rate. Temporal correlation of spike trains was proposed as a potential code for linking the neuronal responses evoked by various features of a same object. In the present study, synchrony strength was measured between cells following an adaptation protocol (prolonged exposure to a non-preferred stimulus) which induce plasticity of neurons' orientation preference.

**Results:**

Multi-unit activity from area 17 of anesthetized adult cats was recorded. Single cells were sorted out and (1) orientation tuning curves were measured before and following 12 min adaptation and 60 min after adaptation (2) pairwise synchrony was measured by an index that was normalized in relation to the cells' firing rate. We first observed that the prolonged presentation of a non-preferred stimulus produces attractive (58%) and repulsive (42%) shifts of cell's tuning curves. It follows that the adaptation-induced plasticity leads to changes in preferred orientation difference, i.e. increase or decrease in tuning properties between neurons. We report here that, after adaptation, the neuron pairs that shared closer tuning properties display a significant increase of synchronization. Recovery from adaptation was accompanied by a return to the initial synchrony level.

**Conclusion:**

We conclude that synchrony reflects the similarity in neurons' response properties, and varies accordingly when these properties change.

## Background

From the primary visual cortex (area 17; V1), neurons acquire sensitivity and selectivity for orientation, motion direction and other visual features as emergent properties [1-3]. In the cat, more than 90% of V1 neurons are well tuned to stimulus orientation [4]. Such orientation selectivity is generally considered a fairly "hard-wired" property acquired before or at the time of eye opening and maintained by patterned visual experience [5]. However, it was reported in the adult cat that V1 neurons could temporarily shift their preferred orientation following prolonged exposure (adaptation) to a non-preferred orientation [6-8] – but see [9]. Plasticity in cat V1 was also reported for adaptation to spatial and temporal frequency [10-12] suggesting that it might be a general property at this level of sensory information processing. In mammalian cortex, tuning properties were also shown to change following adaptation to speed [13,14] and motion direction in MT [9] and V4 [15]. In the present study, we took advantage of this phenomenon to examine how orientation tuning plasticity is related to time-correlated activity in V1 neuron pairs.

Relatively small receptive fields make cells respond to an object's local features, and these individual responses require spatial binding across cortical and visual space as well as binding across types of features [16]. This issue is of particular importance for contour integration, a process that is thought to be mediated by neuronal synchrony [17] – but see [18]. Theoretical studies suggest that the precise synchronization of action potentials represents a potential mechanism for binding [19-21]. Consistent with these theoretical considerations, a body of experimental studies showed that synchronous neural activity is correlated with stimulus properties like coherent motion and similarity [17,22-27]. Furthermore, synchrony was reported to be strong between cells with similar feature selectivity [23,28,29], due in part to specific horizontal connections between cortical domains having similar tuning properties [30,31].

The experiments we report here examine the issue of neural synchrony and its relationship to neurons' tuning properties. To obtain a dynamic view of this relationship, adaptation-induced plasticity was used as a means of producing transient changes of preferred orientation difference among V1 neuron pairs. Precise synchronization between neurons has been expected to dynamically reflect functional similarity of neuronal responses, that is, the closer the tuning properties become following adaptation, the stronger the synchrony. We first examined the result of our adaptation protocol. We then looked at how pairwise synchronization is modulated during adaptation-induced plasticity of orientation tuning.

## Results

We carried out pairwise recordings of multi-unit activity in the anesthetized cat's area 17 (V1). An adaptation protocol consisting in the prolonged presentation of a non-preferred stimulus was applied in order to induce a transient plasticity of the neurons' orientation tuning properties. First, we measured the orientation tuning curve of 89 neurons before and following adaptation, and after a 60-minute period of recovery from adaptation. We then formed neuron pairs and measured the temporal correlation between their spike trains prior to and after adaptation-induced plasticity.

### Adaptation-induced plasticity

**Figure 1:**
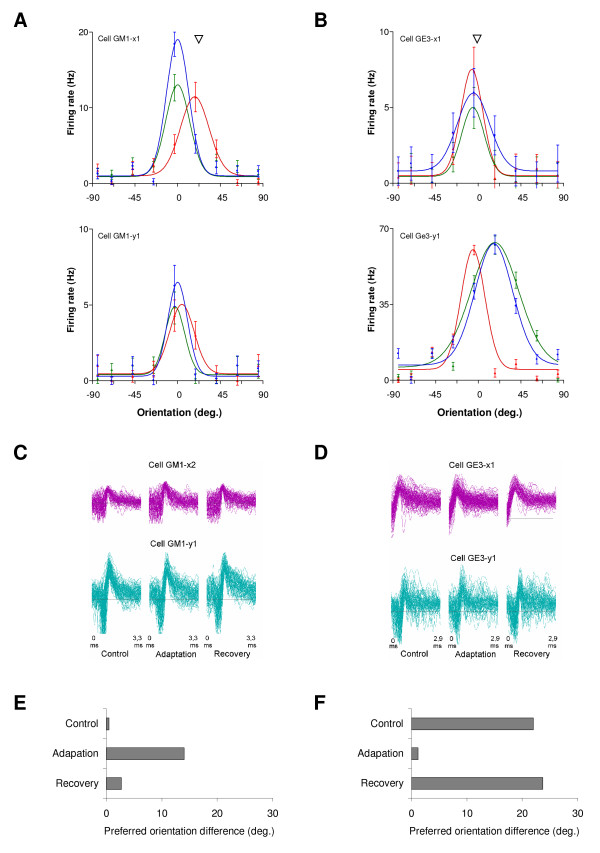
**Adaptation-induced plasticity of orientation tuning in two V1 neuron pairs.** Orientation tuning curves of neuron pairs responding to drifting gratings were recorded in area 17. Curves were centered in relation to the preferred orientation of one cell of the pair in the control condition. Spontaneous activity was subtracted. Arrows indicate the adapting orientation that was presented continuously for 12 minutes. Inter-electrode distance was 400 microns for both pairs. Color code – blue: control, red: adaptation, green: 60 minutes later (error bars denote SEM). (A) Example of an adaptation-induced shift of 18.5° to the right for the cell GM1-x1 and a small shift of 4.5° in the same direction for the other cell GM1-y1. (B) Another example of a 21.9° shift to the left for the cell GE3-y1, but only a very small effect of 1.7° for the other cell of the pair, GE3-x1. (C and D) Respective waveforms for the 2 neuron pairs presented in A and B. The waveforms are similar across conditions, indicating the stability of a cell's activity and discrimination. The S/N ratios were 3.2 and 4.0 for neurons presented in C while S/N ratios of neurons in D were 3.1 and 2.6, respectively. (E and F) The absolute difference of preferred orientation between cells across experimental conditions (A: increase from 0° to 14° after adaptation; B: decrease from 22° to 1.2°). The original preferred orientation difference recovered within 60 min.

Figure 1 illustrates the effect of the adaptation protocol on 2 neuron pairs. The orientation tuning curves of each cell (see Fig. 1A and 1B) are presented for the 3 experimental conditions: control (blue), adaptation (red), and after a period of sixty minutes for recovery (green). For each pair, one cell's preferred orientation in the control condition was used as a reference (centered at 0°). In the first example (Fig. 1A), the adapting stimulus orientation was set at +22.5° (arrow head). The first cell of the pair (Fig. 1A, upper curves) displayed an adaptation-induced plasticity of orientation tuning followed by a recovery of its initial properties. Indeed, this unit's response for the adapting orientation doubled, while its response for the initially preferred orientation (0°) decreased 4-fold. These changes resulted in an apparent slide of the whole tuning curve toward the adapting orientation, an effect that will be referred to as *adaptation-induced shift*, or occasionally in a more concise manner, as *shift *(the latter being distinct from the shift-predictor). Shifts can be classified as attractive or repulsive. An attractive shift occurs when the peak of the tuning curve moves toward the adapting orientation, while a change in the opposite direction is defined as a repulsive shift. Thus the cell in Figure 1A (upper curves) underwent an attractive shift of 18.5° and had recovered its initial preferred orientation 60 minutes after the adaptation protocol was applied. On the other hand, the second cell of the pair (Fig. 1A, lower curves; recorded simultaneously) displayed only a small attractive shift of 4.5°, although the tuning curve peak showed a 30% amplitude decrease. In this example (Fig. 1A), the adaptation-induced shift increased the absolute difference between the optimal orientations of both cells from 0° in control condition to 14.0° after adaptation (see Fig. 1E). Figure 1B shows another example of tuning curves displacement. The adaptation protocol produced a weak shift of 1.7° on the first cell of the pair, but caused a 21.9° attractive shift for the second unit. After adaptation, cells shared virtually identical orientation preference (*Δ *= 1.2°; see Fig. 1F). Recovery of the initial orientation-selectivity properties was observed within sixty minutes. Tests lasted 3 hours on average, a time period during which activity can be lost in electrophysiological recordings for various reasons. It was important in the present study to ensure that the cells forming a pair were responsive and well discriminated during the entire test period. Thus, the stability of the single unit activity waveforms was verified by visual control of the waveforms (Fig. 1C and 1D). In addition, signal-to-noise ratios were calculated for each cell across conditions.

In order to determine the plasticity of orientation tuning in our cell population (n = 89 neurons), curve fits were generated for all cells. The sample size (n = 78) corresponds to the 89 cells that were recorded minus 11 neurons for which we could not obtain a satisfactory curve fit before and after adaptation. In our sample fits accounted for 90% of the variance in the data across conditions.

**Figure 2:**
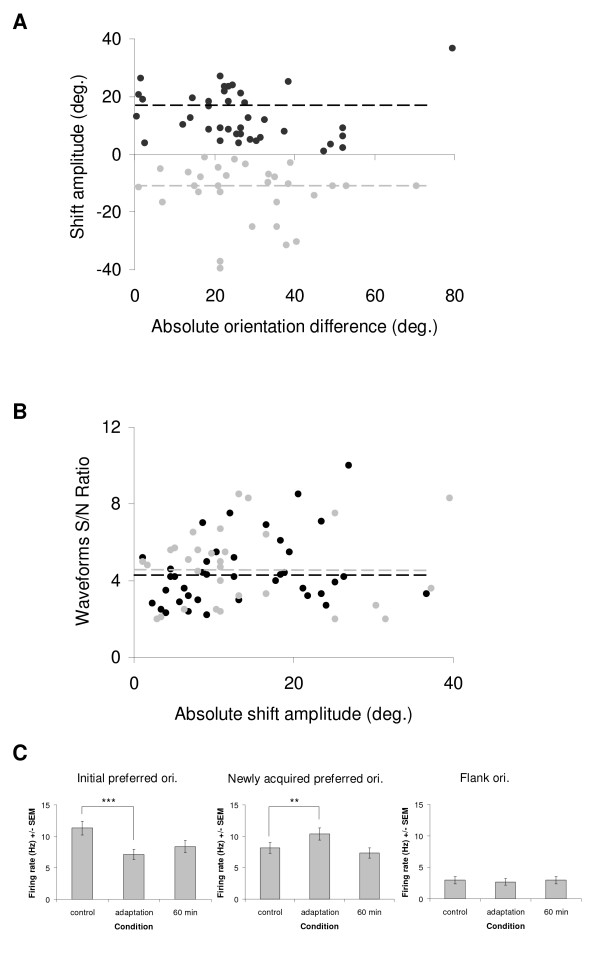
**Adaptation-induced plasticity of orientation tuning in a population of 72 neurons.** (A) Scatter plot showing the amplitude of shifts in preferred orientation after adaptation as a function of the absolute difference between the control preferred orientation and the adapting orientation. Positive values (black dots) designate attractive shifts (n = 42) and negative values (grey dots) designate repulsive shifts (n = 30). The dashed lines in black and grey indicate the mean amplitude for attractive (17.3°) and repulsive (13.5°) shifts, respectively. (B) Scatter plot displaying the signal-to-noise (S/N) ratio of neuronal spikes' waveforms in the control condition as a function of the absolute shift amplitude (black dots indicate attractive shifts, whereas grey dots indicate repulsive shifts). Data are equally distributed around the S/N ratio mean values for both attractive (black dashed line) and repulsive shifts (grey dashed line). This distribution shows that shifts in orientation preference are unrelated to the S/N ratio (r < 0.1 regardless the direction of the shift). (C) Histograms showing the modulation of mean firing rate between *control*, *adaptation *and *60 minutes after adaptation *conditions (error bars are SEM). Left: following the adaptation, a significant decrease of the firing rate is observed for the initial preferred orientation; paired sample two-tailed *t*-test, *p *< 0.001. Middle: in parallel, a significant increase of the response is observed for the newly acquired preferred orientation (attractive and repulsive shifts pooled together); paired sample two-tailed *t*-test, *p *< 0.01. Right: there are no significant changes in the response of far flank orientations (baseline); paired sample two-tailed *t*-test, *p *> 0.1. In all cases, recoveries are shown 60 minutes after the adaptation ended.

The majority of cells (72/78; 92%) displayed a shift in orientation preference. Among those cells, 88% (63/72) showed a significant shift. The scatter plot of Figure 2A shows on a cell-by-cell basis the distribution of shifts as a function of the difference between the cells initial preferred orientation and the adapter. Attractive shifts were observed more frequently than repulsive shifts (58% vs. 42%, respectively). The mean attractive shift was 17.3° ± 2.6° while repulsive shifts averaged 13.5° ± 1.9° (indicated by dashed black and grey lines). In this study, adaptation-induced shifts occurred more frequently for small absolute differences (<45°) between the cell's preferred and adapting orientations, as previously observed [6]. Despite the fact that curve fitting method confers precise preferred orientation in a tuning curve, there is no significant difference in mean shift amplitudes when compared to values obtained from raw tuning curves (paired sample two-tailed *t*-test, p > 0.1). In our sample, neurons were strongly tuned for orientation as revealed by an orientation selectivity index close to 1 (see methods, OSI = 0.80 ± 0.02). Adaptation had no effect on orientation tuning strength. The OSI was almost unchanged after shifts in preferred orientation (0.79 ± 0.02). In Figure 2B, we investigated the potential relationship between the signal-to-noise ratio (S/N) of the spike waveforms and the shift direction and magnitude. Indeed, the waveforms of cells displaying shifts could be noisier (potentially due to spike sorting errors) and thus lead to lower S/N ratios. Conversely, cells exhibiting more stable waveforms would show shifts with smaller amplitudes or even no changes in preferred orientation. Irrespective of the direction of shifts or their magnitude, the S/N ratios are randomly distributed (mean S/N ± SEM = 4.42 ± 0.27 for attractive shifts; 4.71 ± 0.37 for repulsive shifts). This distribution indicates that S/N ratios and shifts in orientation preference are unrelated in the present investigation (r < 0.1 regardless the direction of the shift). The modulation of mean firing rates for the cell population that displayed a shift in preferred orientation is shown in Figure 2C (n = 72). Attractive and repulsive shifts were pooled together to evaluate the increase of response for the new preferred orientation. A significant decrease of responses is observed for the initial preferred orientation after adaptation (left histogram, paired sample two-tailed *t*-test, p < 0.001). Simultaneously, a significant increase of the response occurs for the newly acquired preferred orientation (middle histogram, paired sample two-tailed *t*-test, p < 0.01). However, modulation of responses after adaptation appears to be limited to stimuli around the cells' optimal orientation since there is no significant change in far flank orientations (right histogram, paired sample two-tailed *t*-test, p > 0.1).

### Synchrony modulation through adaptation

**Figure 3:**
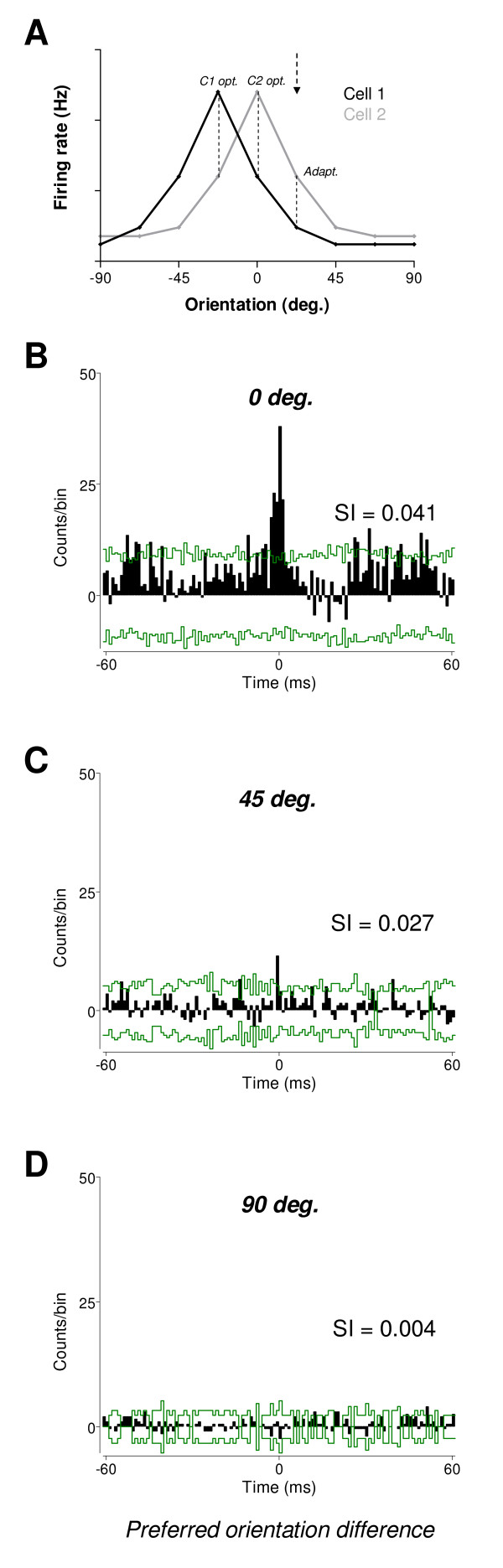
**Synchrony level in relation to the preferred orientation difference in neuron pairs prior to adaptation.** (A) Schematic example of raw tuning curves showing the data points (broken lines) for which the synchrony was measured. In this example the preferred orientation difference is 22.5°. CCHs were computed for the initial preferred orientation of each cell and for the adapting orientation. (B) Example of cross-correlation histogram (CCH) where cells had identical preferred orientation (0°). Synchrony index (SI) measured at 0 time lag, SI value was 0.041. Confidence intervals at 99.9% levels are indicated by green lines. (C) Example of CCH where the preferred orientation difference is 45°. In that case, the SI is lower (0.027). (D) Example of CCH where the difference extends to 90° (rare in our sample). The height of the central peak is clearly not significant being below the upper confidence interval, and the SI value was 0.004. Orientation differences from curves fits measurements was 4.0°, 40.0° and 84.1° in B, C and D, respectively. Pairs comprising neurons with distinct preferred orientations (e.g. in C and D) produced 2 CCHs, only one is shown for sake of clarity.

The schematic example of raw tuning curves in Figure 3A illustrates the data points that were used to construct the cross-correlation histograms (CCHs). We focused our analyses on two cases: when the cells of a pair responded to their respective initial preferred orientation (same or different), and when they responded to an adapting orientation that was common to both cells. It should be emphasized that cells may differ in their preferred orientation, but for each experiment, there was only one adapting orientation. CCHs were generated for each cell's preferred orientation and for the adapting orientation, derived from the raw tuning curves.

To examine the effect of adaptation on pairwise correlated activity, the synchronization index (SI; see Methods for definition) was measured before and following adaptation, and after a 60-minute period of recovery from adaptation. Because the experiments were aimed at measuring the modulation of synchrony in relation to the preferred orientation difference, neuron pairs were selected with respect to two criteria. A pair was kept for analysis if (i) it had a significant SI in at least one condition, and (ii) at least one cell of the pair displayed an adaptation-induced shift in orientation preference on the raw tuning curves. Consequently, from the 103 pairs (89 cells) that were recorded, we selected 52 pairs (60 cells) for further analyses. Among those 52 pairs, 30 comprised cells having different initial preferred orientations for which SIs were computed for each cell's preferred orientation (that is, 60 SI values). The remaining 22 pairs had identical orientation preference, yielding 22 SI values. Altogether, our population amounted to 82 values of SI for the initial preferred orientation, and 52 for the adapting orientation.

Figures 3B, C and 3D display examples of CCHs for 3 neuron pairs showing preferred orientation differences of 0°, 45° and 90°, respectively, prior to adaptation (precise orientation differences calculated from curves fits were 4.0°, 40.0° and 84.1°, respectively). Cells sharing identical orientation preference displayed a large zero lag peak (events in the central bin, see Materials and Methods) in the CCH, and the SI value reached 0.041 (Fig. 3B). In a second example, the preferred orientation difference between the two cells was approximately 45° (Fig. 3C). In that case, the CCH zero lag peak was smaller, and the SI value was 0.027. The CCH of a pair whose preferred orientation difference was large yielded a non-significant zero lag peak, and the SI value was 0.004 (Fig. 3D). In addition, we verified that preferred orientation differences obtained from curve fits approximations agreed with the direct measurements from raw tuning curves prior to and after adaptation. Overall, there is only a weak discrepancy in mean preferred orientation differences using one method or the other (differences less than 2° across conditions, paired sample two-tailed *t*-test, p > 0.05). Thus, orientation differences between raw tuning curves are purposely illustrated in the CCHs examples.

**Figure 4:**
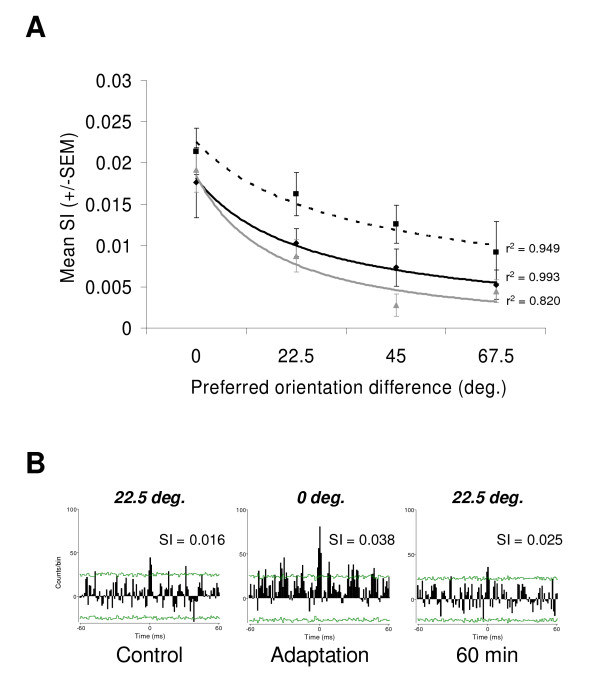
**Synchrony and tuning properties difference.** (A) Relationship between pairwise synchrony and preferred orientation difference in the *control *(continuous black line), *adaptation *(dashed black line) and *60 minutes after adaptation *(continuous grey line) conditions (n = 52 neuron pairs). Error bars denote SEM. Curve fits and respective statistics were added. The general shape of the curves is preserved across conditions. However, the adaptation protocol produced a global increase in mean synchrony, which returned toward control level within 60 minutes. (B) Examples of cross-correlation histograms (CCH) for control, adaptation and recovery. In this particular example, an oscillatory activity emerges after adaptation (T ± 20 ms; 50 Hz). However, CCHs displaying oscillatory temporal structures were rarely observed. For this neuron pair, the control preferred orientation difference from raw curves was 22.5°. Adaptation strongly diminished this difference, and was followed by a complete recovery. Curves fits measurements indicate that the preferred orientation difference for this pair changed from 28.8° to 8.6° following the adaptation and returned to 29.2° after 60 min. Confidence intervals at 99.9% and synchronization indexes are indicated for each CCH.

Figure 4 shows the relation between pairwise synchrony and the magnitude of the preferred orientation difference (n = 52 neuron pairs). The highest SI values were observed for pairs with identical preferred orientations. Pairs with increasingly different preferred orientation (≥ 22.5°) had weaker SIs. A downward trend of the mean SIs was indeed observed as cells displayed greater preferred orientation difference. Curve fits were added for visualization purposes (notwithstanding the goodness-of-fit statistics, 4 data points are not sufficient to elaborate a suitable model). The adapting protocol produced changes in pairs' position along the x-axis. For instance, a pair could move from 22.5° to 0° after adaptation and then return to 22.5° (see Fig. 4B). Such dynamics are not visible in this figure. However, the curves had similar shapes across conditions, meaning that the relationship between synchrony and preferred orientation difference was preserved. Following the 12 min of adaptation a global increase of the SI magnitude was observed (dashed line). Pairwise synchrony returned to control level within 60 min.

**Figure 5:**
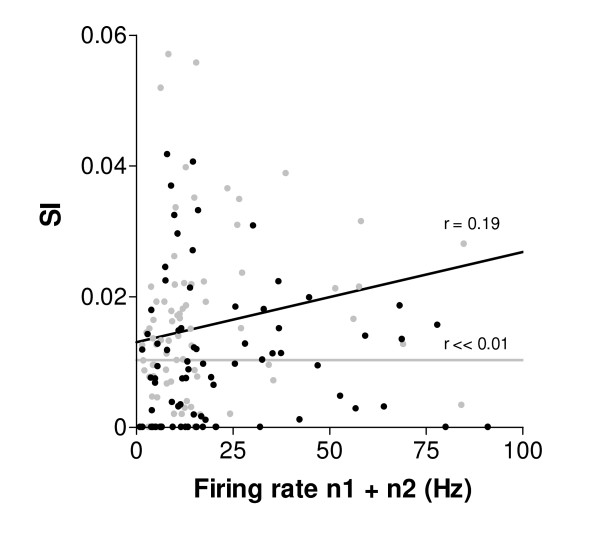
**Firing rate and synchrony strength.** The firing rate of cell pairs was obtained by adding neuronal responses for each initial preferred orientation, n_1 _+ n_2 _(n = 82 firing rate values and corresponding SIs). Linear regressions indicate that there is no relationship between firing rate magnitude and synchrony in control conditions (r << 0.01, grey dots) and only a weak positive one in the adaptation condition (r = 0.19, black dots).

To ascertain that the synchronization index we used does not vary with firing rates, we investigated the relationship between firing rate and synchrony strength, before and after adaptation (Fig. 5). At the population level the mean firing rate of neuron pairs responding to their initial preferred orientations was 24.0 Hz ± 2.8. In control conditions, SI values are unrelated to the discharges of cells at preferred orientations (black dots, r << 0.01). Following adaptation, there is a weak positive relationship between firing rate and synchrony (grey dots, r = 0.19). However, there was no significant difference between the two regressions (Comparison test of two coefficients of correlation, Z_D _= 1.19; p > 0.1). Overall, firing rates did not affect the SI in a significant way, as expected from previous studies [32-34] – but see [35].

**Figure 6:**
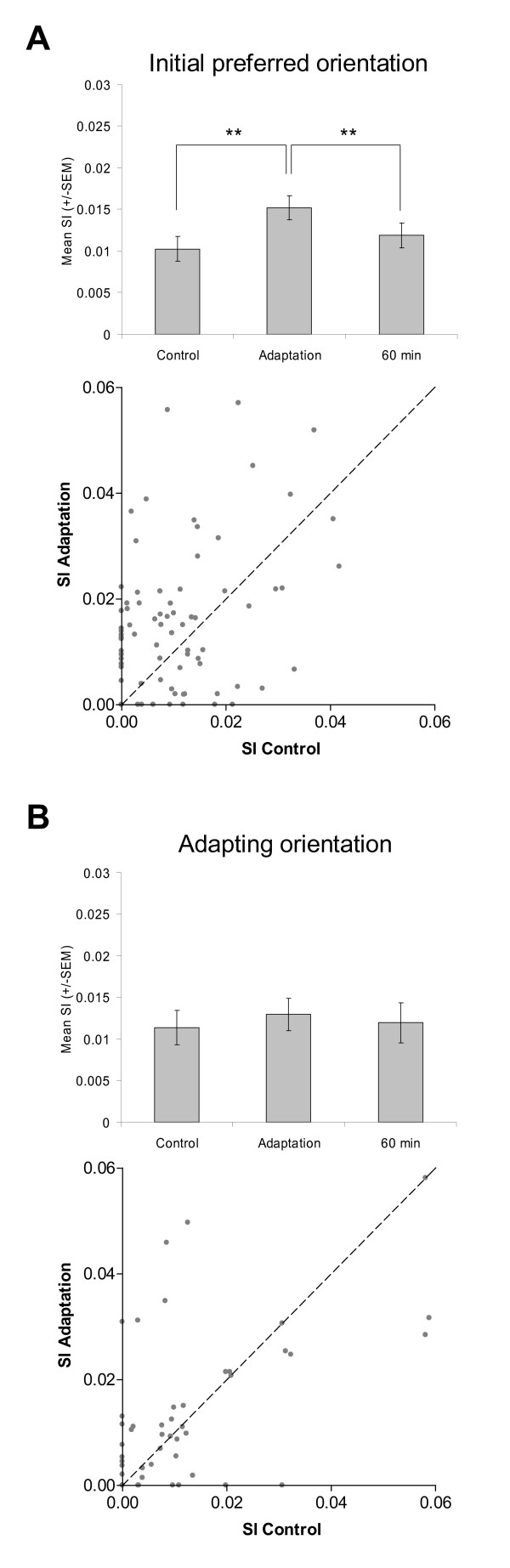
**Mean SI of cells pairs for the initial optimal (n = 82) and the adapting (n = 52) orientation in the three experimental conditions (error bars are SEM).** (A) A significant increase of the mean SI is observed after adaptation for the initial optimal orientation; paired sample two-tailed *t*-test, *p *< 0.001. The underneath scatter plots shows that synchrony increases in 65% of cases, 53/82 SI values are above the equality line (broken line). (B) No changes were observed across conditions for the adapting orientation. The underneath scatter plot indicates that SI values are uniformly distributed along the equality line.

Figure 6 shows the modulation of the pairwise synchrony magnitude for the initial preferred orientation and the adapting orientation across conditions. Correlated activity evoked by the initial preferred orientations stimuli significantly increases (paired sample two-tailed *t*-test, p < 0.001) following adaptation (Fig. 6A). This increase of synchrony was reversible, as after a 60-minute period, the synchronization strength went back to control level. On the other hand, no significant modulation (paired sample two-tailed *t*-test, *p *> 0.1) of synchrony occurred for the adapting orientation across conditions (Fig. 6B).

### Preferred orientation difference and synchrony

**Figure 7:**
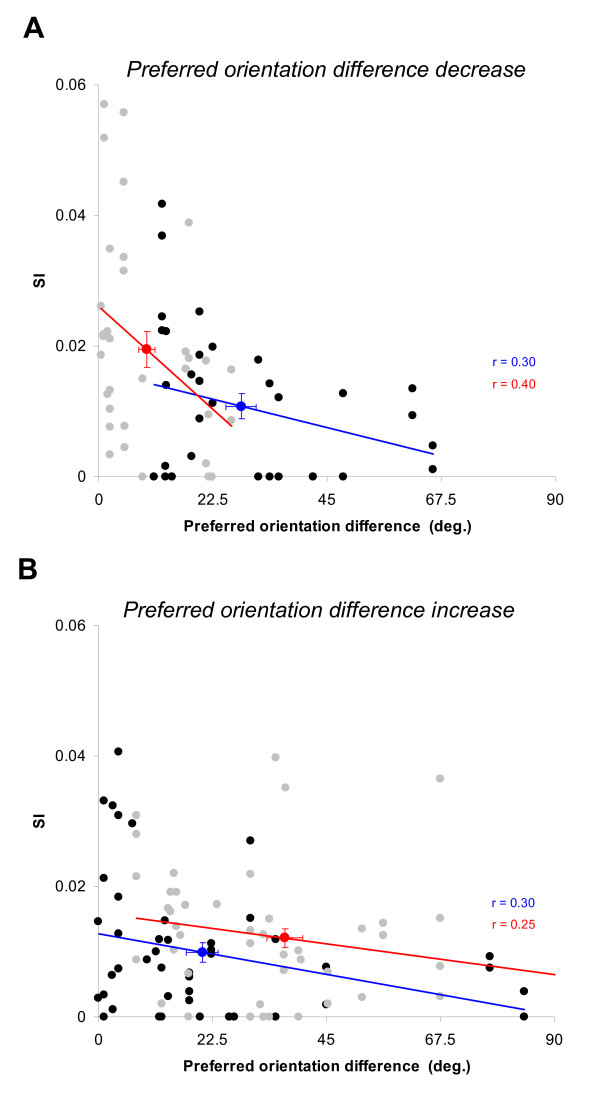
**Relationship between the preferred orientation difference of cells and synchrony strength.** (A) A decrease in preferred orientation difference after adaptation induced a significant rise of the synchrony strength (n = 34 SI values, paired sample two-tailed *t*-test, p < 0.001). (B) An Increase in preferred orientation difference after adaptation induced no significant rise of the synchrony strength (n = 48 values, paired sample two-tailed *t*-test, p > 0.1). The black and grey dots represent the control and the adaptation values, respectively. Linear regressions show that there is a negative relationship between the preferred orientation difference and synchrony in each condition (correlation coefficients are indicated for both group). The bolded blue and red dots correspond to the mean values of preferred orientation difference and the synchrony strength (errors bars in both x and y axis are SEM). Note that the preferred orientation difference was calculated using curve fits. In both case, the preferred orientation difference was significantly different between the control and the adaptation condition (paired sample two-tailed *t*-test, p < 0.0001).

The data presented in Figure 6 was further analyzed to investigate the relation between pairwise synchrony and the adaptation-induced changes of preferred orientation difference in cell pairs derived from the curves fit approximations (Fig. 7). Using curve fits in this analysis gave us the opportunity to evaluate the effect of the adaptation on tuning properties as a continuous function of synchrony strength, rather than arbitrary classification (i.e. orientation difference of 22.5° or 45°). Nevertheless, it must be emphasized that synchrony values were calculated from raw tuning curves for which exact spike numbers are known. The adaptation protocol had 2 effects on the preferred orientation difference (*Δθ*) between neurons. In most cases, the preferred orientation difference increased(*Δθ*_2 _> *Δθ*_1_; 34/52, 65%), one cell shifting away from the other one as illustrated in Figure 1B and 1F. On the other hand, the preferred orientation difference decreased in a significant proportion of cases as well (*Δθ*_2 _<*Δθ*_1_; 18/52, 35%), as illustrated in Figure 1A and 1E.

**Figure 8:**
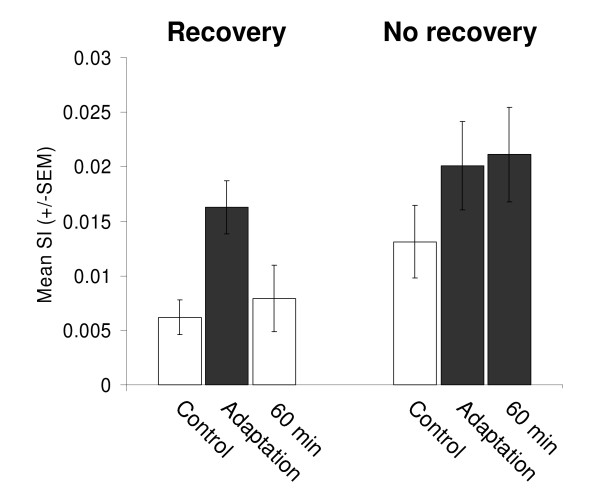
**Comparison of synchrony modulation between neuron pairs that recovered and neuron pairs that failed to recover their initial preferred orientation difference.** Two levels of synchrony were expected, low (white bars) and high (black bars). The low level of synchronization would be associated to the initial preferred orientation difference. The high level would be associated to the newly acquired, smaller preferred orientation difference. To verify our hypotheses, we tested with a nested ANOVA (1) the difference of the means between the 2 groups (F = 14.90, p < 0.001), and (2) the difference of the means within each group (F = 0.37, p = 0.69). The pairs that displayed recovery (n = 18 SI values) had a significant increase of synchrony followed by a full return to control level within 60 minutes. On the other hand, the pairs that failed to recover their initial preferred orientation difference also showed a significant increase of synchrony after adaptation, but that synchrony level remained high 60 minutes after adaptation.

Figure 7 shows how pairwise synchrony was modulated depending on whether preferred orientation difference increased or decreased following adaptation. Remarkably, the pairs of cells whose preferred orientation difference decreased (n = 34 SI values) representing roughly 1/3 of the pairs, contributed in a major way (Fig. 7A). Indeed, only these pairs presented a significant increase of synchronization in the adaptation condition. The mean preferred orientation difference decreases from 28.1° ± 3.0° to 9.5° ± 1.6° while mean SI value doubled (paired sample two-tailed *t*-test, p < 0.001). As expected, there is also a negative relationship between the preferred orientation differences and the synchrony strength, prior to and after adaptation (black dots, r = 0.30 and grey dots, r = 0.40, respectively). In parallel, pairs displaying significant increase in preferred orientation difference showed no significant changes in synchrony strength (n = 48 SI values, paired sample two-tailed *t*-test, p > 0.1). After 60 minutes, the synchronized activity did not return to control level for some of the cell pairs sharing closer preferred orientation, the mean SI value thus remain above the one in control condition (paired sample two-tailed *t*-test, p < 0.1). This observation might suggest a close relationship between synchrony level and preferred orientation difference in neuron pairs. Hence, we adopted a comparative approach that allowed us to test this hypothesis. Figure 8 displays the behavior of the neuron pairs whose preferred orientation difference decreased (Fig. 7A). Those pairs were classified according to whether recovery of initial preferred orientation difference occurred within 60 minutes. In this sub-population, 53% of the pairs (18/34 SI values) recovered their initial preferred orientation difference. We expected 2 levels of synchrony, low (before plasticity) and high (after plasticity).

The low level of synchronization would be associated to the initial preferred orientation difference, and the higher one would be associated to the newly acquired, smaller preferred orientation difference. In the absence of recovery, the high level of pairwise synchrony that was observed after adaptation was expected to persist. In Figure 8, this would correspond to a first group (white bars) with both controls (recovery and no recovery) and the 60 min when recovery occurred, and a second group (black bars) with both adaptations (recovery and no recovery) and the 60 min after adaptation when recovery failed to occur. To verify our hypotheses, we tested with a nested ANOVA (1) the difference of the means between the 2 groups (F = 14.90, p < 0.001), and (2) the difference of the means within each group (F = 0.37, p = 0.69). Our results show that when the pairs displayed a full recovery, the mean SI showed a strong and significant increase after adaptation, and returned to control level. On the other hand, when pairs failed to recover their initial preferred orientation difference, their synchrony increased after adaptation as well but remained high 60 minutes afterwards. We conclude that there is a strong relationship between synchrony level and preferred orientation difference in neuron pairs, and that such a relationship is reflected by the effect of adaptation on both measures.

## Discussion

In the present study, we investigated how the synchronization strength among cortical cell pairs is modified by an adaptation protocol aimed at changing the pairwise preferred orientation difference. In a majority of cells, prolonged presentation of a non-preferred stimulus induced attractive shifts. We also find that synchronization can be dynamically modulated by adaptation-induced plasticity of tuning properties. Indeed, our results show that synchronization between cells becomes stronger when pairwise preferred orientation difference diminishes. In contrast, synchrony is not modulated by adaptation in the cases where the difference between preferred orientations increases.

### Plasticity of orientation tuning

In our sample, most cells displayed a shift in orientation preference following adaptation. Among those cells, attractive shifts were observed more frequently than repulsive shifts (58% vs. 42%, respectively). This proportion is rather different from the ones reported in previous studies in V1. Whereas two groups described mainly repulsive shifts [6,8,36,37], Kohn and Movshon [9] failed to induce shifts of preferred orientation in V1, while the same protocol applied in MT induced attractive shifts. These differences in the adaptation outcome, attractive *vs. *repulsive shifts, are rather intriguing, although an explanation can be found in the various adaptation protocols. First, if we consider the adaptation time, 40 seconds are apparently not sufficient to induce orientation preference shifts in V1 [9], while 2-min adaptation induced mostly repulsive shifts [6], and 12-min adaptation caused a majority of attractive shifts in the present study. Dragoi *et al. *[6] also studied the time course of adaptation and recovery. In their experiments, 3 out of 7 cells in a representative example (see their Fig. 3B and 3C) showed repulsive shifts that were followed during recovery by attractive shifts. These reported 'rebound' attractive shifts had about the same amplitude as the initial repulsive shifts. The time course of these 'rebound' shifts is consistent with the time course of adaptation in our experiments. Thus, an explanation that takes all results into account is that the first effect of adaptation in V1 consists in short-term repulsive shifts and that attractive shifts build up in time. Indeed, recent results showed that adaptation duration from three to twelve minutes reverses the shifts of neurons form repulsive to attractive [38]. Given its duration (adaptation and recovery), our protocol is probably more susceptible to detect attractive shifts in orientation preference. Two other factors may contribute to explain the differences in our results in relation to previous studies in V1: (1) the use by most groups of a "topping-up" protocol, in which the adapting stimulus is presented as a reminder before each test stimulus (2) a possible effect of cortical location and layer [36]. Finally, adaptation to motion direction was shown to induce attractive shifts in area MT. A simple populational model suggests that attractive shifts in MT neurons are consistent with the repulsive shifts in perceived direction observed in psychophysical experiments [9]. Since V1 provides substantial input to MT [39], one interesting question would be to know how tuning shifts in V1 potentially affect or cause shifts in area MT. Overall, our results corroborate the new view of adaptation as an active process including both response depression and enhancement.

### Convergence of orientation tuning properties enhances synchrony

We observed a general increase of pairwise synchrony after adaptation, independently of the preferred orientation difference (Fig. 4A). This effect of adaptation may be related to orientation discrimination. Indeed, cooperation (*i.e. *the advantage gained from the synchronous activity) between V1 neurons is considered as a supplementary channel of information that is crucial for fine discrimination of orientation [40,41].

Adaptation-induced plasticity gave us the opportunity to examine the modulation of cells pairwise synchronization for various preferred orientation differences, by allowing experimental manipulation of such differences. Adaptation (prolonged exposure to a stimulus) can considerably reorganize the boundaries of cortical orientation maps as demonstrated by optical imaging. In adult cats revealed that during adaptation-induced plasticity, orientation preference maps undergo transient changes in the millimeter-order [6]. In the distance range we used, it is likely that cells shifting their preferred orientations toward the adapting orientation also experienced a reorganization of the iso-orientation domains to which they belong. In the case of a cell pair with both cells having the same preferred orientation after adaptation, the two cells might be transiently part of the same iso-orientation domain. Interestingly, our data indicate that time-correlated activity of neurons forced to respond preferentially to the same orientation strongly increases. To a certain extent, that is comparable to the synchronization displayed by neurons belonging to columns with like-orientation preference. Indeed, following adaptation, the synchrony between cells initially belonging to different orientation columns in the control condition seems to emulate the high inter-columnar correlated activity observed between cells with similar tuning properties [23,28,29]. In general, we observed recovery of pairwise synchronization within sixty minutes, as well as recovery of the pre-adaptation tuning properties. However, after a sixty-minute period, some cells were still responding preferentially to the adapting orientation, and were probably still in the same iso-orientation domain, their synchronization thus remaining high. Our results therefore indicate that adaptation-induced plasticity is a reversible process, with variable recovery dynamics from cell to cell.

### Potential mechanisms

The mechanisms underlying orientation selectivity in the primary visual cortex are still debated [42-44]. The earlier models (feedforward) suggested that the selectivity of cortical cells originates primarily from the convergence of lateral geniculate nucleus (LGN) afferences [45,46]. More recent models (recurrent) suggest that the LGN input is broadly tuned and that a sharpening due to lateral inhibitory connections takes place in V1 [47-49]. Although the recurrent models seem to provide the best description of V1 data, both feedforward and recurrent models explain some of V1 neurons properties [44]. Excitatory feedback from higher visual areas like area 21a may also play a role. Chemical activation or inactivation of area 21a was indeed reported to cause major plasticity of area 17 neurons' orientation preference [50]. In adult cortices, plasticity and cortical remodeling mostly originate from higher stages outside of layer IV, the LGN recipient layer [51-54]. Possible loci for plasticity would be layers II and III that involve vertical connections from layer IV, recurrent inputs from other pyramidal cells and/or intrinsic horizontal connections [52]. It was demonstrated that in visual and barrel cortices, long-term potentiation (LTP) of neurons in layers II/III persist beyond puberty [54,55]. Interestingly, in this investigation recordings were performed essentially in supragranular layers (< 1000 *μ*m deep; mean 580 *μ*m ± 70 *μ*m). Adaptation-induced modifications of orientation tuning in mature cortex could thus implicate thalamo-cortical as well as local and long-range cortico-cortical networks connecting neighbouring orientation columns.

Moreover, intracellular studies indicate that, depending on the recorded cell, orientation tuning properties stem from a variety of combinations of excitatory and inhibitory inputs [42,56,57]. The latter could be related to a study by Dragoi et al. [36] where adaptation-induced plasticity of orientation tuning was shown to be loci-dependant: the closer a cell is to a 'pinwheel center' (convergence point of several iso-orientation domains), the more it is susceptible to plasticity [58].

It is likely, although not certain, that the mechanisms involved in adaptation-induced plasticity of orientation preference are the same as the mechanisms causing the pairwise synchrony modulation. Usrey and Reid [59] distinguish 3 categories of cortical synchronous activity: (i) synchrony from anatomical divergence, (ii) stimulus-dependent synchrony, and (iii) emergent synchrony (oscillations). The first category of synchrony is caused by a single source that projects a strong input (feedforward or feedback) onto multiple targets. The constant application of a non-preferred orientation could reinforce thalamo-cortical synapses, and thus synchrony from thalamo-cortical anatomical divergence. However, these connections are weak and need to be synchronized to efficiently drive cortical neurons [60]. Experimental recordings of thalamo-cotical neurons demonstrate the presence of spike patterns suggesting that synchronous spike volleys occur at the population level [61]. If synchronous activity extends across many thalamo-cortical neurons, time-correlated output spikes appear between spiny stellate cells in layer IV [61]. Synchrony in the LGN can also occur via cortico-thalamic projections [62] that may relay the 30–60 Hz rhythm (emergent synchrony) generated by intracortical mechanisms [63]. Even then, thalamo-cortical synapses, which represent ≈ 10% of a cortical cell's total inputs, are unlikely to generate the large changes in orientation preference that were reported in the present investigation. Stimulus-dependent synchrony is what was measured, although some of its components (stimulus coordination) were suppressed in the shift-corrected cross-correlation histograms [64]. Indeed, the shuffling and subtraction procedure (shift-correction) allow the measurement of synchrony of neuronal origin. It was suggested that correlation of V1 single neuron's responses arises for the most part from an orientation-tuned input that causes sharp synchronization [65]. In this investigation, shifts in orientation selectivity and synchrony modulation appear to be related particularly when cells were compelled to share identical orientation properties (see Fig. 7). An intuitive explanation for these findings would imply that adaptation-induced plasticity affects the ascending inputs from layer IV and the horizontal connections which link clusters of neurons displaying identical preferred orientations. Early in life, synapses are extremely plastic and the development of horizontal connections may depend on time-correlated activity triggered by visual experience. In the adult primary visual cortex, synchronous activation selectively stabilizes neuronal connections within and among iso-oriented columns that fine-tune modularity [23,28,29]. Following a prolonged adaptation, pyramidal neurons that displayed closer tuning properties are more coactivated most probably through recurrent reinforcement of their local horizontal excitatory synapses. This supplementary coactivation would enhance the synchrony between clusters of cells as long as they exhibited closer orientation tunings (see Fig. 8). Considering that synchrony and both orientation selectivity and plasticity are thought to occur from intracortical interactions, mechanism involving specific horizontal connections in supragranular layers seems the more suited to explain the simultaneous changes in orientation preference and pairwise zero-lag synchronization.

## Conclusion

We found that in cat V1 orientation-selective neurons, the prolonged (>10 min) presentation of a non-preferred stimulus induces mainly response facilitation for the non-preferred stimulus and depression for the preferred one. This predominance of attractive shifts contrasts with previous similar studies. We propose that the adaptation duration is the major explaining factor: short-term adaptation causes repulsive shifts in V1, but if adaptation is maintained longer, the repulsive shifts are reversed to attractive shifts.

We have also shown that synchrony reflects similarity of tuning properties, specifically orientation preference, and is modulated accordingly when these properties change following adaptation. This novel result suggests a role for neural synchronization in dynamically linking cortical regions with similar functional properties in the presence of their optimal stimulus. Stimulus-dependent synchronization was shown to provide a positive information contribution [66] and might represent a crucial mechanism for efficiently conveying the relevant information to latter stages of visual processing [67,68].

## Methods

### Animals

Fifteen adult cats (2.5–3.5 kg) were prepared for electrophysiological recordings from area 17 (superficial layers) as described in a previous report [69]. Experimental procedures followed the regulations of the Canadian Council on Animal Care as well as the US National Institutes of Health guidelines for the care and use of animals in research, and were approved by the Institutional Animal Care and Use Committee of the University of Montreal.

### Preparation, anesthesia and surgical procedures

Animals sedated with acepromazine maleate (Atravet, Wyeth-Ayerst, Guelph, ON, Canada; 1 mg·kg^-1^, intramuscular) and atropine sulfate (ATRO-SA, Rafter, Calgary, AB, Canada; 0.04 mg·kg^-1^, intramuscular) were anesthetized with ketamine hydrochloride (Rogarsetic, Pfizer, Kirkland, QC, Canada; 25 mg·kg^-1^, intramuscular). Lidocaine hydrochloride (Xylocaine, AstraZeneca, Mississauga, ON, Canada; 2%) was injected subcutaneously as a local anesthetic during surgery. A tracheotomy was performed for artificial ventilation, and one forelimb vein was cannulated. Animals were then placed in a stereotaxic apparatus. Xylocaine gel (Astra Pharma, Mississauga, ON, Canada; 5%) was applied on the pressure points. For the remaining preparations and recording, paralysis was induced with 40 mg and maintained with 10 mg·kg^-1^·h^-1 ^gallamine triethiodide (Flaxedil, Sigma Chemical, St. Louis, MO, USA; intravenous) administered in 5% dextrose lactated Ringer's nutritive solution. General anesthesia was maintained by artificial ventilation with a mixture of N_2_O/O_2 _(70:30) supplemented with 0.5% isoflurane (AErrane, Baxter, Toronto, ON, Canada) for the duration of the experiment. Electroencephalogram, electrocardiogram and expired CO_2 _were monitored continuously to ensure an adequate level of anesthesia. The end-tidal CO_2 _partial pressure was kept constant between 25–30 mmHg. A heated pad was used to maintain a body temperature of 37.5°C. Tribrissen (Schering-Plough, Pointe-Claire, QC, Canada; 30 mg·kg^-1 ^per day, subcutaneous) and Duplocillin (Intervet, Withby, ON, Canada; 0.1 mL·kg^-1^, intramuscular) were administered to the animals to prevent bacterial infection. The pupils were dilated with atropine sulfate (Isopto-Atropine, Alcon, Mississauga, ON, Canada; 1%) and the nictitating membranes were retracted with phenylephrine hydrochloride (Mydfrin, Alcon, Mississauga, ON, Canada; 2.5%). Plano contact lenses with artificial pupils (5 mm diameter) were placed on the cat's eyes to prevent the cornea from drying.

A craniotomy (6 × 6 mm) was performed over the primary visual cortex (including parts of both A17 and A18, Horsley-Clarke coordinates P0–P6; L0–L6). The underlying dura was removed, and once the electrodes were positioned in area 17, the hole was covered with warm agar (3–4% in saline). Melted wax was poured over the agar to provide stability and to prevent it from drying.

### Recording

Multi-unit activity in the visual cortex was recorded by two sets of tungsten microelectrodes (Frederick Haer & Co, Bowdoinham, ME, USA; 10 M at 1 kHz). Each set, consisting of a 4-microelectrode linear array (inter-electrode spacing of 400 *μ*m) enclosed in stainless steel tubing, was controlled by a separate micromanipulator. The signal from the microelectrodes was amplified, band-pass filtered (300 Hz – 3 kHz), digitized and recorded with a 0.05 ms temporal resolution (DataWave Technologies, Longmont, CO, USA). Action potentials were sorted out using window discriminator for further off-line analyses. Multi-unit recordings from one electrode usually included 2 (up to 3) well-isolated single units. The spike sorting method was based on cluster classification in reduced space (Autocut 3.0, DataWave Technologies). Z-scores were computed to quantify the difference between clusters. The stability of each cell's activity across conditions was verified qualitatively by visual control of the clusters disposition and of the waveforms shape (see Fig. 1C and 1D). The signal-to-noise (S/N) ratio was measured as the mean of the waveforms amplitude divided by the noise in the last bin of the temporal window (range: 1.9 to 3.4 ms).

### Visual stimulation

Stimulation was monocular (dominant eye). After clearly detectable activity was obtained for 2 microelectrodes on one of the arrays, the multi-unit receptive fields (RF) were mapped as the minimum response fields [70] by using a hand-held ophthalmoscope. Eye-screen distance was 57 cm. RF edges were determined by moving a light bar from the periphery toward the center until a response was elicited. These preliminary tests revealed qualitative properties such as dimensions, velocity preference, orientation and directional selectivity. To ensure that both electrodes did not record spikes generated by the same cells, only microelectrodes from the same array were used for the analysis, because precise inter-electrode distances could not be guaranteed between the two electrode arrays. In our study, the interelectrode distance (400 to 1200 *μ*m) was within the range of receptive fields overlapping for area 17 in cats (5mm^2^) [71]. Accordingly the majority of recorded neurons had overlapping receptive fields. Visual stimuli were generated with a VSG 2/5 graphic board (Cambridge Research Systems, Rochester, England) and displayed on a 21-in. monitor (Sony GDM-F520 Trinitron, Tokyo, Japan) placed 57 cm from the cat's eyes, with 1024 × 768 pixels, running at 100-Hz frame refresh. Stimuli were sine-wave drifting gratings covering both RFs [72,73]. Contrast was set at 80%. Mean luminance was 40 Cd.m^-2^. Optimal spatial and temporal frequencies were set within the 0.2–0.4 cycles·deg^-1 ^and 1.0–2.0 Hz range respectively, where V1 neurons are known to respond well to sine-wave drifting gratings [74]. In the first step, orientation tuning curves (16 equidistant points covering 360°, i.e. by steps of 22.5°) were determined using a single grating covering both RFs. Nine orientations covering 180° and centered on the preferred orientation (and direction) of one site were then used for the rest of the experiment. Each orientation was presented in blocks of 25 trials, with each trial lasting 4.1 s and a random inter-trial interval (1.0–3.0 s). Thus, recording sessions lasted for 25–30 min (25 trials*(4.1s + 2s) for each of 9 oriented-stimulus). Orientations were presented in random order. Peri-stimulus time histograms were recorded simultaneously for both sites. It should be noted that these tuning curves were obtained for moving stimuli, so it is strictly speaking incorrect to describe them as orientation tuning curves. Indeed, orientation is by definition cyclic over the interval 0°–180°, while direction is cyclic over the interval 0°–360° [75]. In other words, for any given orientation, there are 2 possible perpendicular directions for a moving stimulus. Considering that most cells in the cat visual cortex show some degree of direction selectivity [1,76], a proper description of their responses would rather be a directional tuning curve. However, this distinction will be ignored, as it has been in almost all other studies of orientation tuning in V1 [75].

Following the tuning properties characterization, an adapting stimulus was presented continuously for 12 minutes. The stimulus was a drifting grating whose orientation was generally set 22.5 to 45.0° off the preferred orientations of both sites (see arrows in Fig. 1A and 1B). No tests were conducted during this adaptation period. Immediately after adaptation, the orientation tuning curves of both sites were determined once again. In order to exclude effects which may arise from different randomization sequences during the post-adaptation recordings, responses to the adapting stimulus were always measured first followed by 3–4 semi-random orientations around control preferred stimuli. Hence, the most critical tested orientations were measured within 10–15 min following adaptation. Other orientations were further tested in random order. This procedure was adopted for all cells in order to insure robust effect of the long-term adaptation on near flanks of cells' preferred orientation. In addition, it should be mentioned that responses at far flank orientations (baseline) remained constant during recordings (see Fig. 2C). A last recording was performed 60 minutes post-adaptation to assess the stability of the tuning properties, i.e. the recovery time course. A recording session lasted 3 hours on average.

Preliminary tests with various adaptation and recovery times were conducted (data not shown). In our experimental conditions, an adaptation period of 12 minutes appeared sufficient to induce a shift in orientation selectivity. Longer time intervals were tested for recovery. A 60-minute period seemed a good compromise since the neurons' activity could be lost over the course of the experiment for longer durations. Within this time window, recovery of the initial properties was observed for about one half of the sites. No significant difference was observed in the recovery course whether the animals were left unstimulated or stimulated with randomly-oriented drifting gratings.

### Data analysis

Once single cells were sorted out off-line from multi-unit spike trains accumulated during data acquisition, the cells from both electrodes were paired, and cross-correlation histograms (CCHs) were constructed (1-ms binwidth). To examine synchronization of neural origin, stimulus-induced coordination (i.e. time-locked responses) and rate covariation had to be removed. To that effect, shift-predictors were computed by correlating spike recordings shuffled by two stimulus presentations, and these were subtracted from the raw CCHs [64]. All subsequent analyses were performed on the shift-corrected CCHs. Repeated shuffling allowed us to calculate the 99.9% confidence limits, which correspond to 3.3 standard deviations in a normalized distribution. Only peaks exceeding the confidence limits were considered statistically significant [77]. Synchronization strength was computed as a correlation coefficient [78-80]. This correlation coefficient, or synchronization index (SI), reflects the strength of time-correlated activity in a neural CCH as a function of the number of simultaneous events normalized in relation to the firing rate of each neuron. As a consequence, the synchronization strength is considered independent of the response levels, i.e. the mean firing rates.

The synchronization index is defined as

(1) SI=[CE][(N1−(N1)2/T)⋅(N2−(N2)2/T)]12,

where CE is the number of coincidental events in the central bin of the shift-corrected CCH, and N_1 _and N_2 _are the total number of discharges recorded simultaneously from two neurons during time T (4,096 ms × number of trials) [79]. The bin size (1 ms) allows the measurement of the time-correlated activity within a 1-ms time window (zero-lag synchronization). Orientation tuning curves were analyzed in two ways. First, for the precise measurement of adaptation-induced shifts in orientation preference (Fig. 2), curve fits were generated using the von Mises function:

(2) *M*(*θ*) = *A*.*e*^*b*(*cos*(*θ*-*c*))^+*d*

where A is the value of the function at the preferred orientation, c, and b is a width parameter. An additional parameter, d, represents the spontaneous firing rate of the cell [9,75]. A fit was considered satisfactory if it accounted for at least 80% of the variance in the data. To ensure that our cells were properly tuned for orientation, we used an orientation selectivity index (OSI). It was measured using the fitted tuning curves, by dividing the firing rate at the baseline (orthogonal orientations) by the firing rate for the preferred orientation, and subtracting the result from one [81,82]. The closer the OSI is to 1, the stronger the orientation selectivity. To test the significance of tuning shifts curve fits using von Mises function were generated on cells responses for every presentation (n = 25, see details above). Then, we compared between trial by trial the preferred orientation obtained prior to and after adaptation. A *t*-test revealed the significance level [6]. Cells showing shifts in preferred orientation larger than 5° were statistically significant (paired sample two-tailed *t*-test, *p *< 0.01). The curve fitting method is the appropriate way of estimating the preferred orientation in a tuning curve and thus shifts in orientation selectivity [75]. However, notwithstanding the gain in precision in comparison to raw tuning curves, the resulting optimal orientation would be located between the actual data points, a location for which there are no spike trains recorded. Although interpolation is an option, there is to our knowledge no indication of its physiological pertinence. Consequently, raw spike counts were used for all analyses involving synchrony calculations.

## Authors' contributions

NG and AN carried out all experiments, performed analysis and wrote the manuscript. SS participated significantly in all phases of the investigation. SM designed the study and contributed in the in the manuscript redaction. All authors read and approved the final manuscript.
